# A biochemical screening platform to target chromatin states using condensates as a tool

**DOI:** 10.1016/j.slasd.2025.100236

**Published:** 2025-04-28

**Authors:** Laura J. Hsieh, Tracy Lou, Muryam A. Gourdet, Emily Wong, Geeta J. Narlikar

**Affiliations:** a Department of Biochemistry and Biophysics, University of California, San Francisco, San Francisco, CA 94158, USA; b Tetrad Graduate Program, University of California, San Francisco, San Francisco, CA 94158, USA; c TippingPoint Biosciences, Inc., San Francisco, CA 94107, USA; d Lead Contacts, USA

**Keywords:** Chromatin, Condensates, PPIs, IDRs

## Abstract

Chromatin states define cell fates and consequently dysfunctional chromatin states drive disease. Conventional approaches to target dysfunctional chromatin states typically rely on targeting a defined, structured binding pocket of a specific chromatin protein. However, drugs developed from targeting single chromatin proteins have often failed in the clinic due to toxicity from broad non-specific effects on the genome. Substantial previous work has indicated that the function of a given chromatin state is encoded in the context-dependent protein-protein interactions (PPIs) between the Intrinsically disordered regions (IDRs) and folded domains of the multiple constituents. Currently, there are no drug discovery approaches that target the complex multivalent protein interactions within a given dysfunctional chromatin state. Therefore, new methods are required to target chromatin within specific conformational contexts for better translation into humans. Prior discoveries from our group and others have shown that chromatin intrinsically forms condensates through weak, yet specific, multivalent interactions between itself and other components. Using this intrinsic property of chromatin, we have developed a new screening method to address this technology gap and identify modulators of dysfunctional chromatin states for drug discovery. Here, we show that we can recreate different chromatin contexts as phase-separated condensates that have distinct biochemical and biophysical properties. Furthermore, we have scaled the technology into a screening platform and identify small molecules that modulate chromatin states specifically based on their chromatin context. We anticipate that such specific targeting of a disease driving chromatin assembly would reduce off-target effects, translate better into humans and open a new landscape of therapeutic possibilities for targeting complex, multivalent interactions.

## 1. Introduction

Conventional target-based drug discovery programs rely on biochemical assays for single proteins with defined binding pockets. However, in cells much of the proteome is found in crowded multicomponent assemblies where the components interact dynamically [1]. Targeting a factor in isolation thus lacks crucial biological context and can yield drugs that are either ineffective in vivo or that also affect healthy cells, resulting in toxic side-effects [2]. Additionally, *>*50 % of the proteome is composed of proteins that have at least one intrinsically disordered region (IDR), with over 30 % classified as intrinsically disordered proteins (IDPs) [3]. Thus, much of the proteome is unstructured and does not have well-defined pockets making them undruggable by conventional approaches. Importantly, IDRs are critical for generating and imparting specificity to the dynamic multi-component assemblies within which much of biological processes occur [1]. Their conformational flexibility allows IDRs to adopt different conformations when interacting with different structured domains [4]. While IDRs interact with their target proteins with high specificity dictated by their specific sequences, their interactions are of low affinity due to their highly dynamic nature [1]. IDRs are especially enriched in nuclear proteins that modify DNA packaging (chromatin) in eukaryotic cells [1], which raises the possibility that successfully drugging of IDRs may be a broadly useful strategy for targeting diseases originating from dysfunctional chromatin states.

Chromatin is fundamentally packaged into nucleosomal units which involves wrapping of ~ 150 bp of DNA around an octamer of histone proteins. Most nucleosomes are decorated with post-translational modifications and bound by additional protein components to impart specific functions to given regions of the genomes. Such assemblies are collectively referred to as a chromatin state. Chromatin states that contain histone modifications such as acetylation and more loosely packed nucleosomes enable active gene transcription and are called euchromatin [5]. In contrast, chromatin states that contain specific types of histone methylation and have more closely packed nucleosomes function to repress genes and are called heterochromatin [5]. In addition to regulating gene expression, chromatin states also regulate DNA damage, DNA recombination and nuclear integrity [5]. In all these processes the specific composition of a chromatin state and the network of interactions within the state determines how each process is regulated. An aberrant composition rewires the interaction network resulting in defects in gene regulation, DNA replication and DNA repair with the resultant dysfunction leading to disease [6,7]. In fact, changes to chromatin states in many diseases result in only a small detectable change in transcriptional programs, suggesting that changes in chromatin states impact cell function and identity through broader effects beyond transcription [6].

With increasing knowledge about the roles of aberrant chromatin, genome-wide studies are revealing that many diseases become resistant to treatment because of structural changes in chromatin rather than changes in DNA sequence [6]. At the same time as more data on chromatin structure from patients comes to light, aberrant chromatin appears to be a general driver for multiple disease pathologies. In the laboratory, manipulating chromatin using molecular biology tools is very effective at stopping, slowing or even reversing disease [6,7]. However, converting the knowledge gained from molecular biology to developing effective and safe small molecule-based therapies targeting aberrant chromatin has been difficult. Classical epigenetic drugs like HDAC inhibitors or methyltransferase inhibitors have been successful but only in certain types of blood cancers [8]. Their broader efficacy has been limited for two main reasons: development of resistance to the drug and cumulative side-effects [8]. This is where targeting chromatin factors in the appropriate context becomes critical. The challenge arises because most chromatin factors are found broadly across the genome and possess multiple different functions. Therefore, to achieve better specificity and higher efficacy in manipulating aberrant chromatin, we need newer approaches that target complete aberrant chromatin states rather than individual factors out of context.

We and others have shown that chromatin intrinsically forms condensates through weak, yet specific, multi-valent interactions between the different components [9-11]. We have also found that the key factors that drive formation of a major type of heterochromatin do so by using their IDRs to form phase separated chromatin condensates in vitro and in vivo [10,12]. In vitro, these heterochromatin condensates can be modulated by known biological regulators that affect chromatin interactions in vivo [13]. More broadly, condensates formed in vitro with core components can recapitulate the key hallmarks of the corresponding in vivo chromatin states [9-12]. Thus, the condensate behavior of chromatin now allows us to recreate specific physiologically or disease relevant chromatin states in a test-tube to screen for drugs that modulate an entire chromatin state. Using condensates as a tool we have developed a technology that screens for small molecules that specifically disrupt disease-driving interaction networks in an aberrant chromatin state without affecting the integrity of physiological chromatin states [14]. This method for the first time allows the targeting of chromatin factors within a specific biological context, which we anticipate will improve the specificity of drugging pathological chromatin. Further, targeting whole chromatin states also allows us to exploit differences in material properties between chromatin states at the mesoscale. We describe our new approach in this technological brief below using examples of a heterochromatin state mediated by the protein HP1α and a euchromatin state mediated by the protein Brd4.

## 2. Materials and methods

### 2.1. F40 sortase purification

Recombinant F40 sortase was expressed from BL21(DE3) cells and purified as previously described [15].

### 2.2. Histone purification and semi-synthesis

All histones were expressed from BL21(DE3) pLysS cells and purified as previously published [16,17]. To make native modifications on H3, H3Δ32 was purified and the H3 semi-synthesis was performed as previously described [15] with the following modifications: 80 uM H3Δ32 histone was mixed with 1.2 mM H3K9me3 or H3K27ac depsipeptide (aa1–31) (GenScript), and 300 uM F40 sortase in 50 mM HEPES (pH 7.5), 150 mM NaCL, 5 mM CaCl_2_, and 1 mM DTT at 37 °C for 18 h. The pellet was resuspended in 20 mM Tris (pH7.8), 1 mM EDTA, 7 M urea, 10 mM NaCl, and 5 mM BME and run over a HiTrap Q HP in tandem with a HiTrap SP HP column, and eluted off the HiTrap SP HP. The ligated H3 with the modification was confirmed using LC/MS and by Western blot with antibodies recognizing the modifications (Abcam).

### 2.3. S1000 column preparation

The Sephacryl S1000 super fine resin slurry (Cytiva) was mixed and added into an empty XK 26/600 column (Cytiva) and residual liquid was drained at room temperature until the liquid covered the resin. This was repeated until the resin reached the top of the column. The column was hooked up to an FPLC and 1 CV of water was run on the column at a flow rate of 3 ml/min to pack the resin until the resin had fully settled. After the first round of packing, more resin was added and packed at 3 ml/min until the resin settled at the top of the column. A total of ~750 mls of S1000 slurry was needed to fully pack the column.

### 2.4. 12 × 601 array DNA purification and labeling

Plasmid containing the 12 × 601 array DNA [11] was propagated in Stbl2 cells (NEB) and was purified using the GigaPrep Kit (Qiagen). To isolate the 12 × 601 array DNA, the plasmid was digested with restriction enzymes EcoRV and XhoI (NEB) at 37 °C for 18 hours, and purified by gel filtration using a custom packed S1000 column. The DNA was subsequently ethanol precipitated and eluted in 1X TE.

Array DNA was cut with XhoI to create a 5′ overhang for fluorescent nucleotide labeling with Klenow Fragment 3′ – 5′ exo- (NEB). Purified array DNA (1mg/mL) was incubated with the Klenow fragment (0.032U/μg), 35μ M dATP, dTTP, dGTP, Alexa Fluor 647-aha-dCTP (ThermoScientific), and 1X NEBuffer 2 overnight at room temperature, covered in foil. Labeled DNA was subsequently purified by phenolchloroform extraction, followed by an additional ethanol wash, and then resuspended in 1X TE at 4mg/mL.

### 2.5. Chromatin assembly

Histones were refolded in high salt buffer to form octamer and purified by size exclusion chromatography as previously published [16]. Nucleosome arrays were assembled using salt gradient dialysis as previously described [10]. Chromatin assembly was evaluated by digesting arrays with EcoRI and running the digested chromatin on a 6 % native gel.

### 2.6. Brd4 purification

The short isoform of Brd4 (BRD4S) was expressed and purified using previously described methods [18]. Brd4 was labelled with AF-488 dye using labeling KIT (ThermoFisher). Brd4 was labelled in a Tris-free media. Using a Tris buffer reduces labeling efficiency because the AF-488 dye labeling KIT (ThermoFisher) uses a tetrafluorophenyl (TFP) ester moiety. TFP esters are highly reactive with primary amines, which exist in Tris buffers. Post labeling, BRD4 was dialyzed in buffer containing HEPES.

### 2.7. HP1α purification

Human pBH4-HP1α was transformed into Rosetta BL21 (DE3) competent cells in *E.coli*. Cultures were grown at 30C in 2XLB with 50μg/ml carbenicillin and 25μg/ml chloramphenicol until OD ~ 0.4 was reached. Cells were then transferred to 18C and grown to OD ~ 0.8 before inducing expression with 0.4 mM isopropy-βD-thiogalactopyranoside for 16 h. Cells were harvested by centrifugation at 4000 g for 30mins and resuspended in lysis buffer (1X PBS, 300 mM KCl, 10 % glycerol, 7.5 mM imidazole, and protease inhibitors – 100 mM phenylmethanesulfonyl fluoride, 2 μg/ml Aprotinin, 3μg/ml Leupeptin, and 1 μg/ml Pepstatin A). The cells were then lysed using the C3 Emulsiflex, and clarified lysate was obtained by centrifugation at 25,000 g for 30mins. Clarified lysate was incubated with Talon cobalt resin for 1 h at 4C with rocking. The resin-lysate solution was added to a gravity column and washed with ~50mls lysis buffer without protease inhibitors and eluted with 10mls elution buffer (20 mM HEPES pH 7.2, 100 mM KCl, 400 mM imidazole). Protein was dialyzed overnight at 4C with 0.5 mg TEV protease/L culture in dialysis buffer (20 mM HEPES pH 7.2, 150 mM KCl, 3 mM DTT) to cleave off the 6x-His tag and to remove imidazole. The cleaved protein was then injected onto a Mono-Q 10/100 GL anion exchange column (GE) and eluted with a 150–800 mM KCl gradient over 15 column volumes. Clean fractions were pooled and concentrated in an Amicon Ulta 10 K spin concentrator before final injection onto a Superdex S75 Increase 10/300 GL column running with size-exclusion/storage buffer (20 mM HEPES pH 7.2, 300 mM KCl, 10 % glycerol, 3 mM DTT). Desired fractions were pooled, concentrated, and flash frozen in liquid nitrogen for long term storage at −80C.

### 2.8. HP1α labeling

pBH4-HP1α was modified with a “GSKCK” tag at the C-terminal end, and cysteine 133 was mutated to a serine to encourage specific labeling. HP1α-ΚCK was purified following the HP1α purification method. Before labeling, the protein was dialyzed in labeling buffer overnight (20 mM HEPES pH 7.2, 350 mM KCl, 10 % glycerol, 0.5 mM TCEP) at 4C. Protein concentration was then adjusted to 200μ M and mixed with Cy3 maleimide at a 1:1 molar ratio. The reaction was immediately quenched (~5 secs) with 10X molar excess β-mercaptoethanol. Free dye was separated from labeled protein using illustra G50 column following manufacturer’s protocol. Labeled protein was then flash frozen in liquid nitrogen and stored at −80C. Labeling efficiency was determined by comparing Cy3 dye absorption to HP1α concentration. 150,000M– 1cm– 1 at 552 nm was used for Cy3, and 29,495 M – 1cm– 1 at 280 nm was used for HP1α.

### 2.9. In vitro droplet assay (Fig. 4)

HP1α was diluted to desired 2X concentration. HP1α ΚCK was added to HP1α at a ratio of 1:500 HP1α: HP1α ΚCK for evident fluorescent signal. HP1α-chromatin droplets were formed at 40 nM nucleosome array concentration in 20 mM HEPES pH 7.5, 0.1 mM EDTA and 2X HP1α-HP1α KCK in 20 mM HEPES pH 7.2, 150 mM KCl 1 mM DTT. 10μ L of 80 nM nucleosome arrays (2X) was mixed with 10μl 2X HP1α-HP1α ΚCK and incubated at room temperature for 20 min before transferring to a glass bottom 384 well plate for imaging.

Brd4S was diluted to desired 2X concentration. Brd4S AF488 was added to Brd4S at a ratio of 1:50. BRD4S-chromatin droplets were formed at 30 nM nucleosome array concentration in 20 mM HEPES pH 7.5, 0.1 mM EDTA and 2X Brd4S-Brd4S-AF488 in 20 mM HEPES pH 7.5, 150 mM KCl 1 mM BME. 5 μL of 120 nM nucleosome arrays (4X) was mixed with 5μl 4X phasing reaction buffer (20 mM HEPES pH7.5, 300 mM KCl, 4 mM MgCl2) and 10μl Brd4S 2X and incubated at room temperature for 60 min before transferring to a glass bottom 384 well plate. Droplets were incubated in 3the 84 well plate for 2 hour at room temperature prior to imaging.

384 well glass bottom plates (Greiner Sensoplate 781,892) were prepared for sample examination as follows:

The wells were rinsed three times with 100μl water, incubated with 100 μl of 2 % Hellmanex for 30mins-1 h, rinsed with water three times again, incubated with 100μl of 0.5 M NaOH for 30 mins, rinsed with water three times, and then 70μl of 20mg/ml mPEG-silane dissolved in 95 % EtOH was added to coat the wells. The plate was covered with foil and left overnight at 4C.

After the overnight, the wells were rinsed with 95 % EtOH five times, followed by incubation with 100mg/ml BSA for 2 h. Then, the wells were rinsed three times with water and three times with 1X phasing buffer afterwards (20 mM HEPES pH 7.2, 75 mM KCl, 0.05 mM EDTA, 0.5 mM DTT). After, the condensates are added to each well.

### 2.10. Screening with small molecules

To conduct droplet assays at scale, arrays assembled with 540μ g fluorescently labeled array DNA and 1.3 mg of fluorescently labeled HP1α were needed for one 384 well plate. 384 well plates were washed and coated in mPEG-silane in bulk using the Biotek EL406 washer dispenser. HP1α-array droplets were screened against a Bioactive compound library containing 320 molecules (Selleck Chemicals). These compounds were diluted in 1X phasing buffer (20 mM HEPES pH 7.2, 75 mM KCl, 0.05 mM EDTA, 0.5 mM DTT), and added to the wells for a final concentration of 10μ M in the reaction. The droplets and compounds were dispensed in the 384 wells using the Biomek-FX.

### 2.11. Microscopy and image analysis

**Fig. 1.**
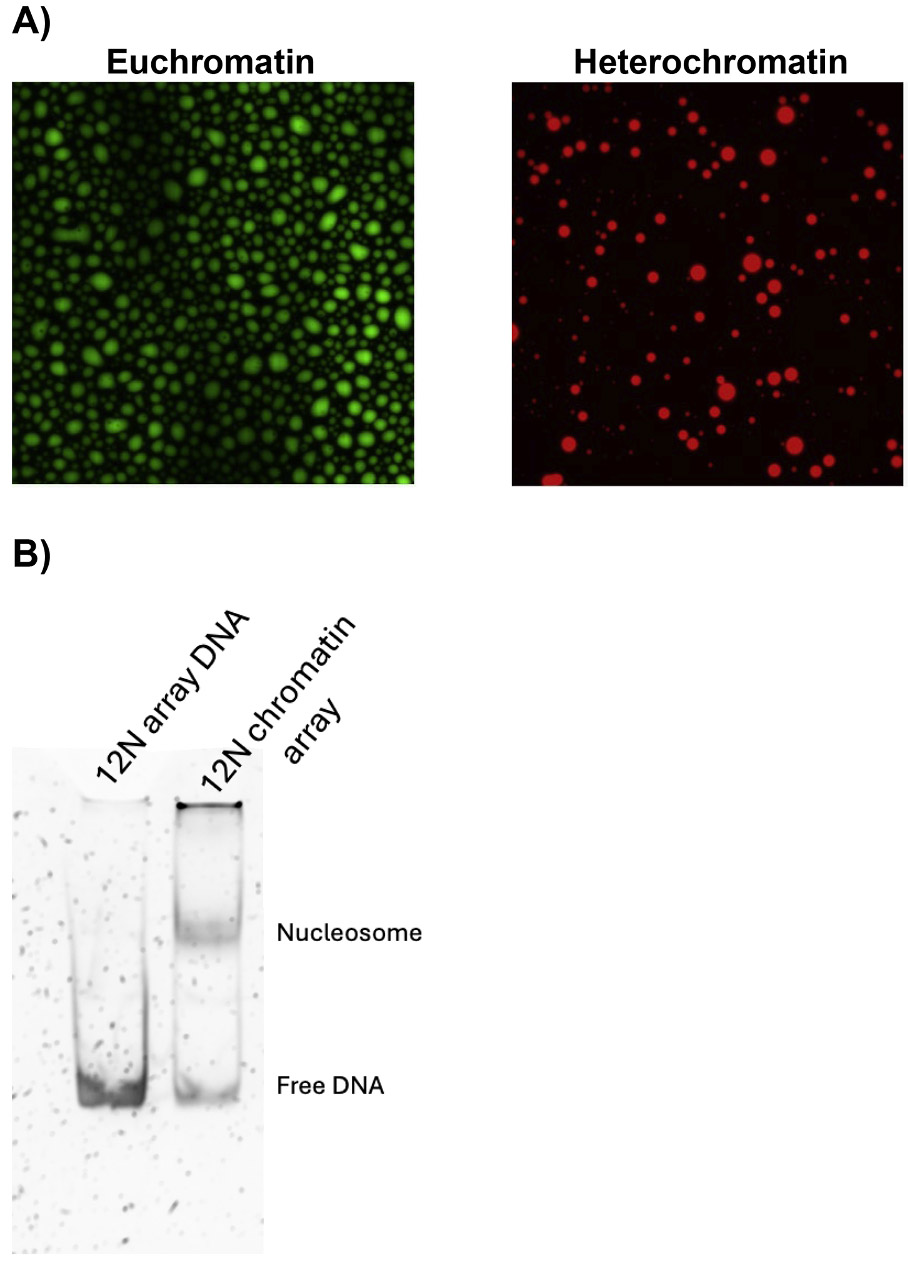
Heterochromatin and euchromatin condensates display different properties. A) On the left and colored in green, euchromatin condensates are formed with BRD4S and H3K27ac chromatin. On the right and in red, heterochromatin condensates are formed with HP1α and H3K9me3 chromatin. The shape, number and surface tension of the droplets differ between these two chromatin states. B) A 6 % native gel showing digestion of 12 N chromatin array compared with digestion of the DNA template alone. Nucleosomes are assembled on the 12 N chromatin array used in the chromatin assays.

After 3–4 h of settling to the bottom of the plate, the condensates were imaged on the InCell Analyzer 6500HS equipped with a Nikon Plan Apo 40x/0.95NA objective and sCMOS camera. Five field of views per channel were taken per well. Image analysis was conducted using a custom pipeline written in Python using NumPy and SciPy SciKit libraries. Classical image segmentation and quantification techniques were implemented to identify droplets and characterize their geometry, fluorescent intensity, and consequently the effectiveness of the small molecules on the droplets. Briefly, images containing fluorescence signals of labeled chromatin arrays are pre-processed by resizing and their background signals removed. Since the visible features of interest are typically much larger than a single pixel, no information is lost and processing can be done faster on smaller images. The background signals typically only have low-frequency signal (slowly-varying) which is effectively removed by the "rolling-ball" algorithm [19]. Size distribution is computed from the pre-processed images by successively opening [20] the input image with increasing area thresholds while keeping track of the total peak intensities lost due to area opening in each iteration [21]. No fixed footprint is used in opening the image and thresholding is not explicitly performed. Image opening produces "flat" peaks whose area equals the specified value and their peak heights lower than the original peak (Fig. 1). The surface areas used for opening the input image are calculated as areas of a circle with increasing diameter sizes. While in theory the process can be repeated until no intensities remain, stopping the algorithm to a specified maximum size is enough to obtain sufficient information and saves some computation time. To incorporate a global characteristic of the input image, the y-axis is scaled by the average intensity of the input image.

Size distributions are information-rich but are not practical in a high-throughput screen. To reduce the complexity of the size spectrum, standardized moments are computed from the size distributions. While no single number alone can sufficiently describe the entire size distribution, a few numbers comprising of higher-order moments (e.g. up to 4th-order, describing kurtosis of the distribution) can uniquely describe the size distribution. The equations for computing the moments are: first moment (mean)

μ = ∑ x * y * Δ_x_ second moment (variance)
σˆ2 = Σ (x - μ)ˆ2 * y * Δ_x_
(standardized) third moment (skew)
Σ ((x - μ)ˆ3 / σˆ3) * y * Δ_x_
(standardized) fourth moment (kurtosis)
Σ ((x - μ)ˆ4 / σˆ4) * y * Δ_x_

Prior to computing these moments, y is normalized such that Σ y * Δ_x_ = 1. x is the diameter sizes used for opening. Area threshold is computed as 2π*(x/2)ˆ2. Δ_x_ is the spacing between diameter sizes.

### 2.12. Cell culture and imaging

NIH3T3 cells were grown on fibronectin-treated coverslips in DMEM Glutamax supplemented with 10 %FBS at 37 o C. Cells were passaged into media containing either 1 % DMSO or 10 μM small molecule in 1 % DMSO, blind coded TP SM1-SM5. Cells were treated for either 3 hrs or 2 days, then stained for H3K9me3 (Abcam Ab8898), HP1α (EMD Millipore MAB3584) and DNA (Hoescht). Cells were imaged on a Nikon CSU-22 spinning disk confocal equipped with a Evolve Delta EMCCD camera (Photometrics) and manually scored for morphological abnormalities. Cells were first globally screened at 40x, subsequent to high-resolution imaging at 100x.

## 3. Results

### 3.1. Mesoscale properties of condensates change based on chromatin components

To exploit the use of chromatin condensates as a drug discovery tool, we first sought to determine whether changes in chromatin composition result in mesoscale changes that can be visualized in chromatin condensates. To this end, we recapitulated distinct chromatin states, formed condensates and visualized the condensate behavior via fluorescently labeled chromatin. We formed two types of chromatin states, one representing euchromatin and one representing heterochromatin. For the euchromatin state we used chromatin assembled with histones that were acetylated on histone H3 at lysine residue 27 (H3K27ac) and the protein BRD4S. Both the acetylation mark and the BRD4S are often core components of euchromatin. A domain termed the bromodomain in BRD4S recognizes the acetyl marks on histones. To model heterochromatin states we used chromatin assembled with histones that were trimethylated on histone H3 at lysine residue 9 (H3K9me3) and the protein HP1α. Both the H3K9me3 mark and the HP1α are core components of heterochromatin. A domain termed the chromodomain in HP1α recognizes the H3K9me3 mark. The specific chromatin modifications representative of the respective chromatin hallmarks, H3K9me3 for heterochromatin and H3K27ac for euchromatin, were engineered using a sortase-based ligation method [15]. Additionally, for comparison we also investigated condensate formation by BRD4S and HP1α with unmodified chromatin. In all cases, the chromatin was represented by an array of 12 evenly spaced nucleosomes (12 N Array, see Materials and Methods). This array was incubated with either BRD4S or HP1α, mixed and plated to form condensates (see Materials and Methods). We found that the mesoscale properties of the condensates were different based on the composition of the chromatin state. These changes could be visualized and quantified microscopically (Fig. 1). Specifically, we scored the droplets on the following features: fluorescence intensity, condensate size, number of condensates and shape.

Interestingly, the BRD4 condensates with H3K27ac chromatin were less circular compared to the HP1α condensates with H3K9me3 chromatin, suggesting the BRD4S containing condensates have a lower surface tension (Fig. 1). Lower surface tension could reflect weaker interactions within BRD4-chromatin condensates compared to the HP1α- chromatin condensates. BRD4 condensates with H3K27ac chromatin also formed larger droplets compared to the HP1α condensates with H3K9me3 chromatin (Fig. 1). The larger droplets could reflect higher miscibility and more liquid like behavior for the BRD4-chromatin condensates compared to the HP1α- chromatin condensates.

**Fig. 2.**
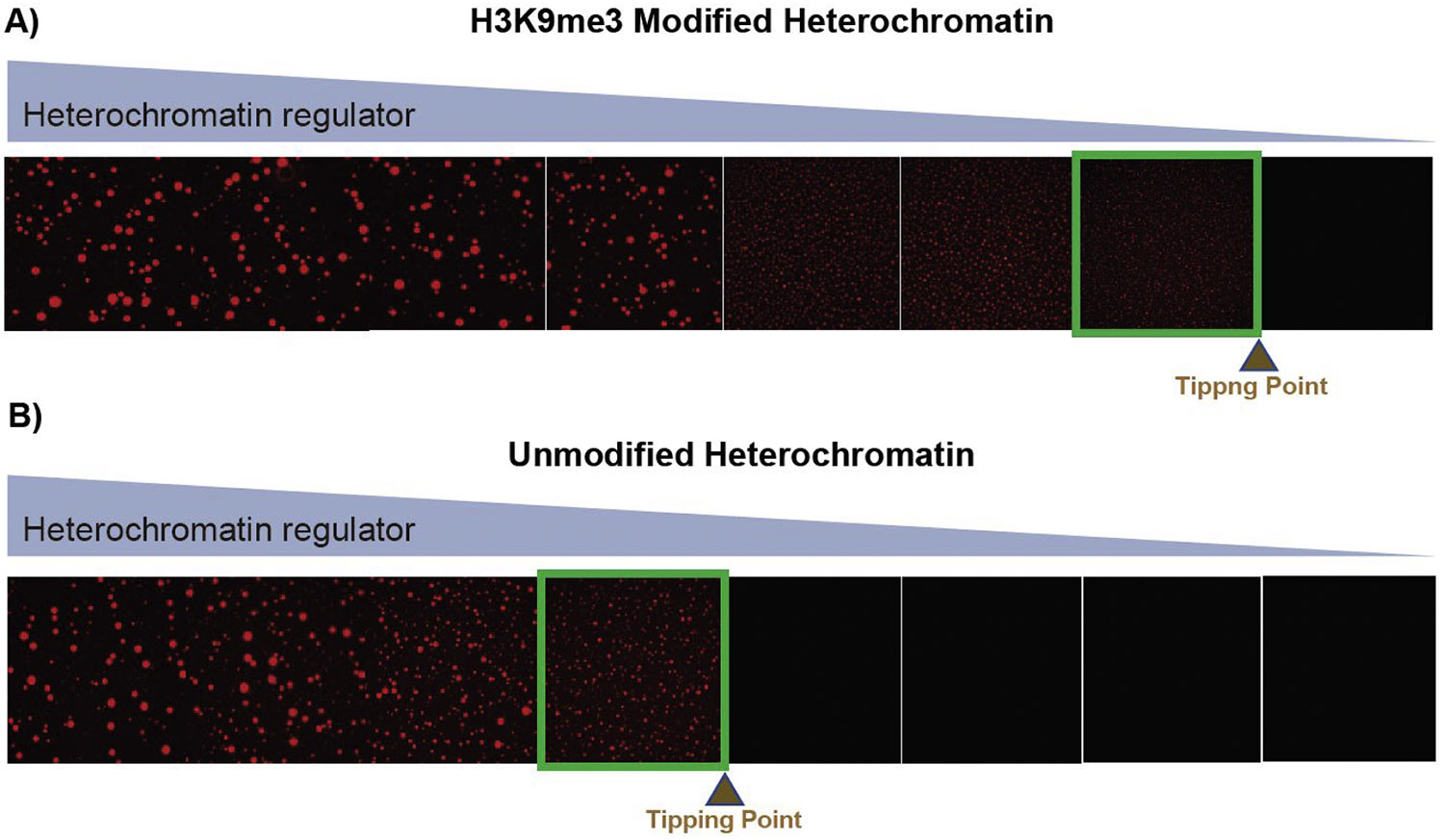
Dysfunctional HP1α heterochromatin states are less robust and more easily tipped over to being dissolved. A) Heterochromatin condensate with the proper H3K9me3 modification forms condensates in the presence of increasing concentrations of HP1α. The critical concentration of the interactions breaking is shown as the tipping point, which in this case is at a specific concentration of the chromatin regulator. B) Dysfunctional heterochromatin condensates that lack the histone modification (unmodified) with titrated amounts of HP1α. The tipping point of breaking the interactions occurs at a much higher level of HP1α with the unmodified H3 than H3K9m3.

Because epigenetic marks have been shown to be indicative of establishing distinct chromatin properties in vivo, we sought to also determine whether a single chromatin modification alters the mesoscale properties of the chromatin condensates. Indeed, altering post-translational modifications on histones had noticeable effects. Specifically, the presence of the heterochromatic H3K9me3 mark lowered the saturation concentration to form condensates by ~3 fold when titrating in HP1α (Fig. 2). These results suggests that our approach captures differences between chromatin states at the mesoscale that can be targeted as a whole.

### 3.2. Small molecules can alter chromatin condensate specifically

**Fig. 3.**
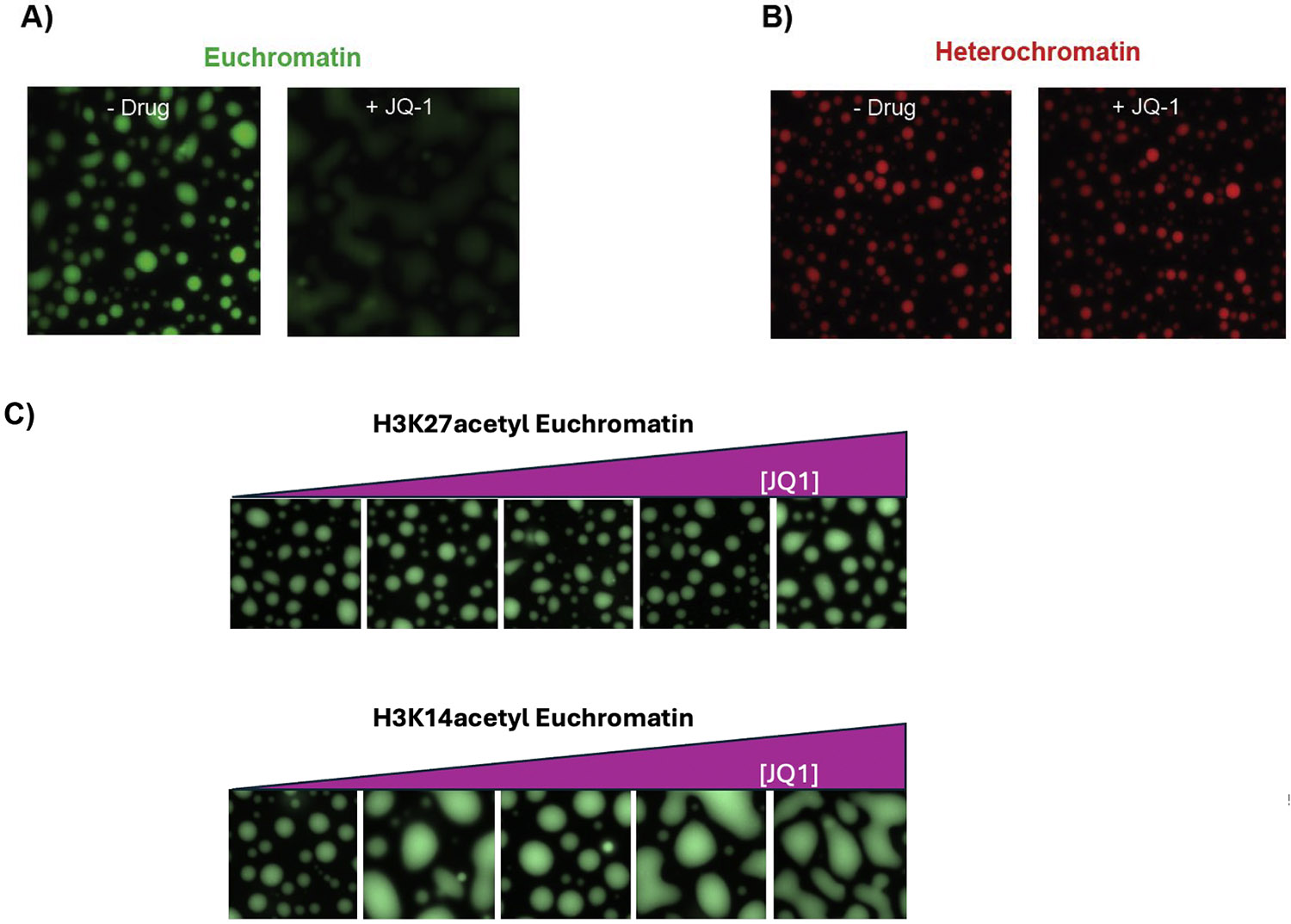
BRD4 euchromatin state is specifically disrupted by BRD4 inhibitors. A) BRD4 euchromatin condensates are shown in green. The left panel is with no drug and the right panel is with 40uM JQ-1 BET inhibitor. Addition of JQ-1 disrupts the BRD4 and Xenopus H3K27ac euchromatin droplets. Decrease in fluorescence intensity of Euchromatin state with the addition of JQ1. B) HP1α heterochromatin condensates are shown in red. The left panel is no drug and the right panel is with 40uM JQ-1. JQ-1 does not bind to HP1α, so there is no effect on heterochromatin condensates. C) JQ1 shows specificity for disrupting BRD4 condensates containing human H3K14acetyl over human H3K27acetyl chromatin arrays. Condensates were generated with 2.5 μM BRD4 and 20 nM 12 N chromatin array containing either human H3.3K27acetyl or H3.3K14acetyl marks. JQ1 concentrations are: 0 μM, 0.10 μM, 1 μM, 5 μM, and 50 μM.

We next investigated whether a small molecule known to target a specific chromatin component in isolation can also target this component specifically within a condensate. Towards this goal we tested a known molecule, the bromodomain (BD) inhibitor JQ1, which is a well-studied inhibitor of BRD4, that binds with nanomolar affinity. We added JQ1 to BRD4 condensates with H3K27ac chromatin condensates and the HP1α condensates with H3K9me3 chromatin. We found that JQ1 dissolves BRD4-chromatin condensates (as shown previously [11]). We further found that this effect of JQ1 is specific as the molecule does not perturb the HP1α -chromatin condensates even up to 40 uM (Fig. 3). These results suggests that the specificity of JQ1 is maintained within the complex environment of a chromatin state. However, what was remarkable was the substantially lower efficacy of JQ1 in the context of a chromatin state. Despite JQ1 being a potent nM binder of BRD4 alone [22], the lowest concentration of JQ1 needed to dissolve the BRD4-chromatin condensates was 40uM. The lower efficacy may arise from the dense network of inter-molecular interactions made by BRD4S that JQ1 must dissolve before binding. The low efficacy BRD4 binding within chromatin condensates may also explain in part the low efficacy of BD inhibitors like JQ1 seen in the clinic [22]. Another explanation for the lower efficacy is the specific acetylation mark. In cells BRD4 localizes with both H3K14acetyl and H3K27acetyl chromatin. Although BRD4 doesn’t acetylate H3K14, recognition of H3K14acetyl by BRD4 promotes acetylation of other H3 and H4 residues by BRD4, including H3K27 [23]. We find that JQ1 disrupts BRD4 condensates formed with human H3K14acetyl chromatin at a much lower concentration than those formed with H3K27acetyl chromatin (Fig. 3C). This data is consistent with previous work implying that BRD4 localization at H3K27acetyl chromatin does not rely on the interaction of the bromodomains with the H3K27acetyl mark. Importantly, this experiment indicates that our condensate-based platform can recreate the distinct chromatin interactions found in cells in a manner that allows for the specificity of action of drugs such as JQ1.These results raise the possibility that a small molecule inhibitor developed against a single factor could be further optimized by structure activity relationship (SAR) within condensate assays to facilitate binding in the context of disease-relevant chromatin states. In addition, it is also possible that more potent and specific molecular scaffolds can be discovered by initiating with a screen that captures effects on chromatin states. With this goal in mind, we leveraged the condensate properties of chromatin states to develop a novel screening platform as described below.

### 3.3. Scaling and enabling a chromatin condensate screening platform

**Fig. 4.**
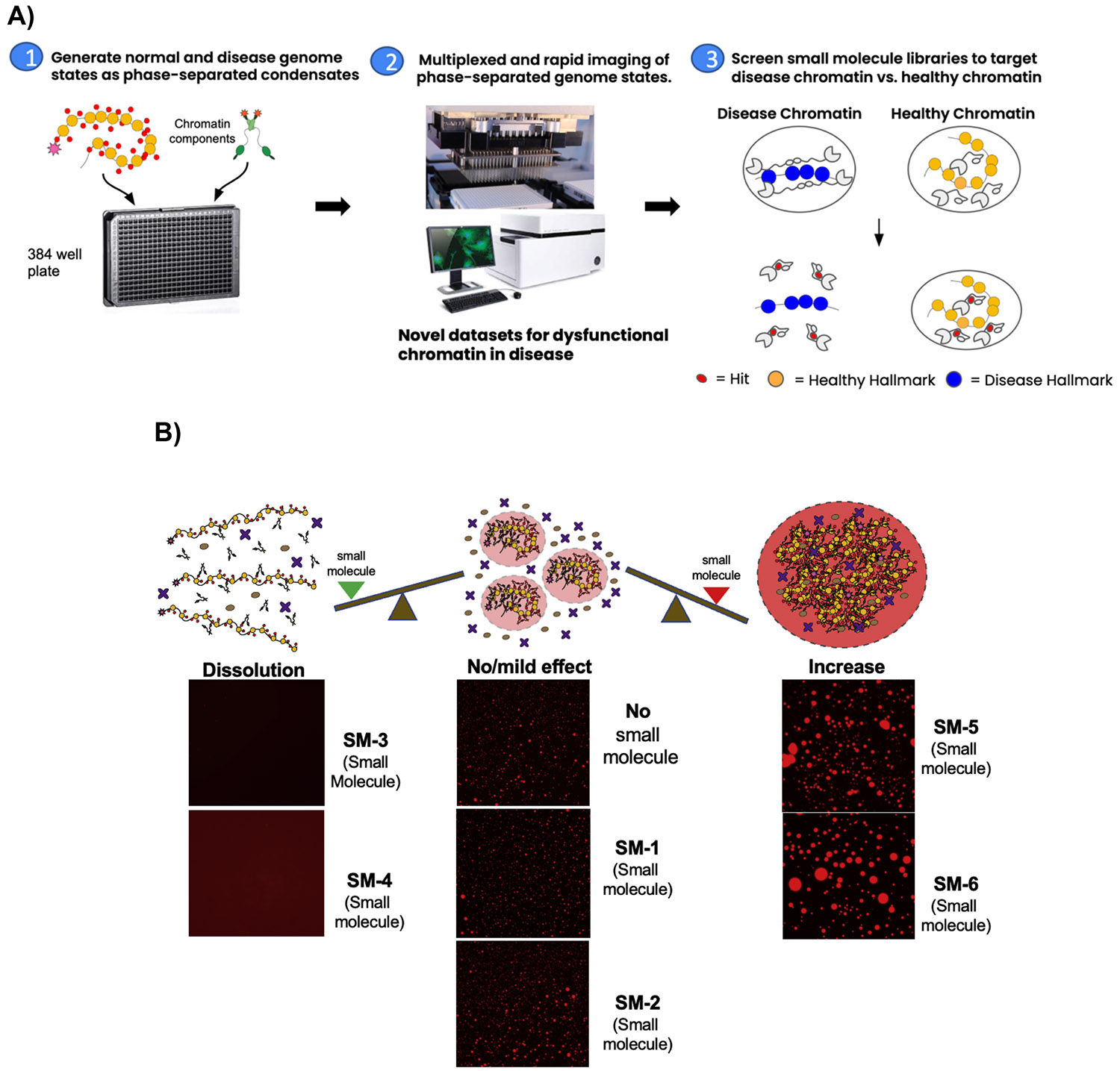
Schematic representation of chromatin condensate screening technology [14]. A) Workflow for screening pipeline. B) Examples of phenotypic changes seen in the presence of small molecules. The small molecules were classified based on changes in the following factors: Change in fluorescent intensity, change in droplet size, and shape.

To increase the throughput to enable screening of chromatin condensates, we scaled up our chromatin assembly and assay platform (as described in Materials and Methods). This included both purification of high amounts of chromatin-associated proteins and chromatin and optimization of our protocols to most efficiently use the purified materials. As a test case, we started with condensates formed by HP1α bound to chromatin containing the H3K9me3 mark. A library of 320 bioactive small molecules was used and the condensates were imaged on an InCell microscope. Using condensate integrity as a measure of changing chromatin protein-protein interactions (PPIs), we screened for small molecules that specifically disrupt the HP1α-chromatin condensates (Fig. 4). For the screen, each small molecule was distributed into one well containing α-chromatin condensates and multiple images within a well were analyzed. The platform has been further optimized and scaled to now screen up to 10,000 molecules at a time (data not shown).

### 3.4. Identification of small molecule hits that specifically alter HP1α heterochromatin

From the screening, we successfully identified small molecule hits that either dissolve or enhance the HP1α -chromatin condensates (Table 1). The vast majority (151 molecules) of the small molecules did not affect condensates. However, there were 84 molecules that partially or fully dissolved condensates, while 81 molecules enhanced condensates. There were also 4 small molecules that affected specific properties of condensates such as alterations in shape or surface tension. Some of these molecules caused the droplet to aggregate into gel-like fibers (data not shown). The others resulted in surface tension changes, causing the droplets to lose rigidity and circularity (data not shown). Altogether, these data show that our platform can be used as a screening tool to identify small molecules that affect the network of interactions in a chromatin state.

### 3.5. Small molecule hits translate into mouse cells

**Fig. 5.**
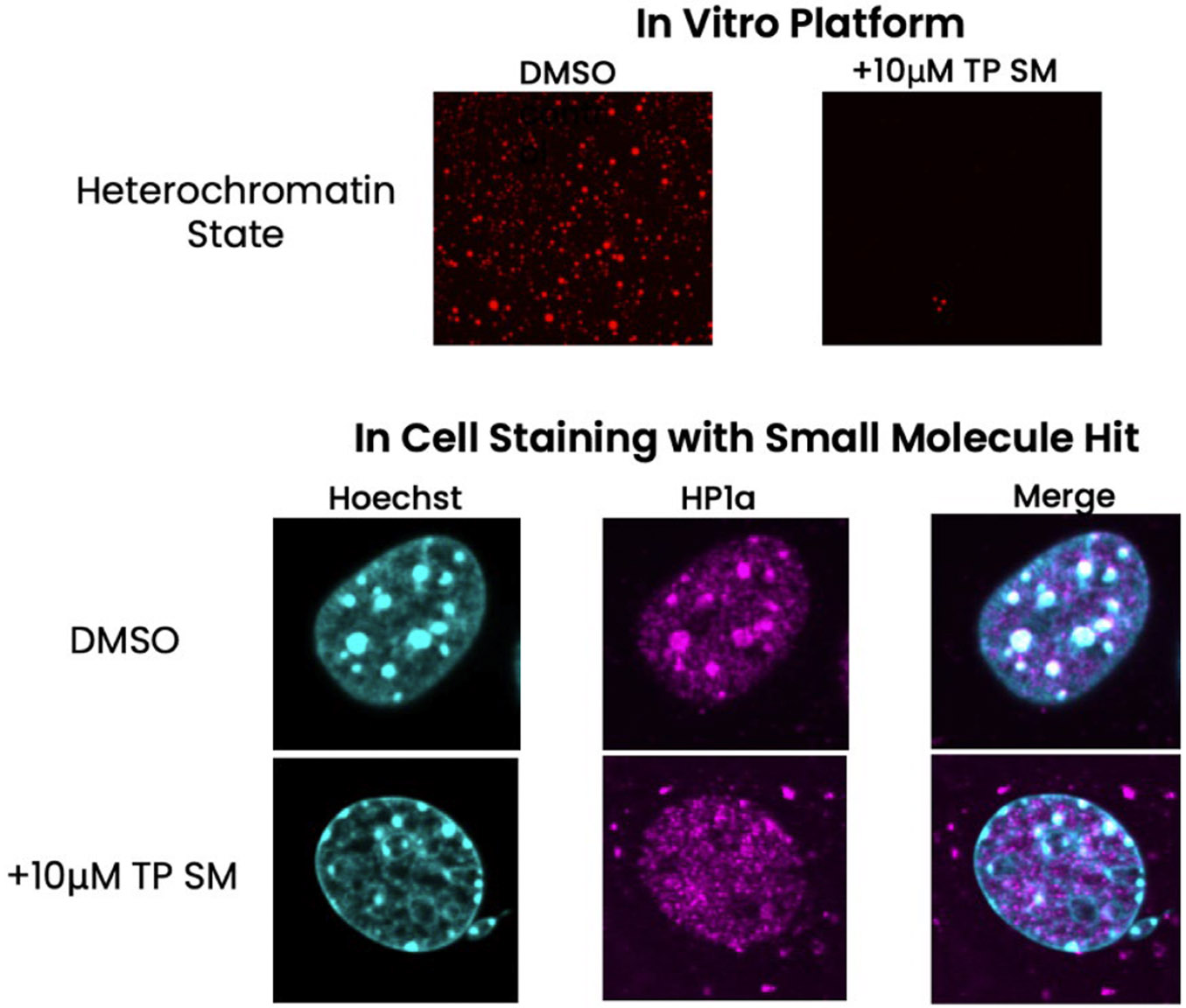
Application of small molecule hits to mouse cells. On the top panel, we show an example of a small molecule hit that was identified off of the condensate platform. This small molecule disrupts HP1α-chromatin condensates at 10uM concentration. On the bottom panel, we test this small molecule at 10uM concentration and the DMSO control in mouse cells and stain with Hoechst in cyan (right) and HP1α in magenta (middle). The merged images are shown on the left.

To validate that our small molecule hits can alter chromatin states in cells, we tested 5 of the small molecules hits that disrupted HP1α from our platform in mouse cells. These 5 hits were first confirmed as positive hits on the platform (*n* = 3 at 10uM concentration). After incubation of 10uM of small molecule in mouse cell lines (*n* = 2 replicates), we observed noticeable qualitative changes in HP1α puncta and DNA staining (example of small molecule shown in Fig. 5). Analogous to our condensate assay, one of the small molecule hits disrupted HP1α puncta and disrupted heterochromatin formation (Fig. 5). In the untreated cells, HP1α mostly co-localized with DNA puncta, but in the treated cells the DNA and HP1α co-localization is reduced. Interestingly, the treated cells also showed nuclear blebbing, suggesting changes in nuclear integrity, likely resulting from changes in HP1α-chromatin interactions. Altogether, our results suggest that the small molecules identified from our condensate platform correlates with small molecule behavior in affecting chromatin alterations in cells.

## 4. Discussion

Here we have shown that we can biochemically reconstitute chromatin states that possess different mesoscale properties that are dependent on specific chromatin marks. Importantly, we demonstrate that subtle perturbations at the single protein level can have significant effects on mesoscale properties that can be visualized using condensates. We also demonstrate the feasibility of screening small molecule libraries with our reconstituted chromatin condensates and identify hits that modulate chromatin states in different ways. Furthermore, we demonstrate that hits generated from the reconstituted condensate platform do indeed affect chromatin structure in cells. This screening approach [14] allows for leveraging the contextual differences in chromatin states for small molecule optimization in a high-throughput, cost-effective manner to enable better specificity in targeting dynamic chromatin states. This technology has now been scaled to accommodate the screening of thousands of molecules and is fully customizable to create different chromatin states. The approach can now be used to 1) separately recreate different types of chromatin states in a test tube, and 2) target the IDR-mediated protein-protein interactions within specific chromatin states.

Our method has two advantages over conventional biochemical screening approaches. First, we can now target an entire disease-causing chromatin state by assaying for small molecules that alter defective chromatin states without disrupting physiological chromatin states. In cells, we anticipate that disrupting the aberrant interactions within a chromatin state can have therapeutic effects through several mechanisms. For example, disruption of chromatin states could activate tumor suppressor and apoptotic genes that are silenced by aberrantly formed heterochromatin. Such disruption could also allow chromatin factors concentrated in disease-associated chromatin to redistribute towards more physiological interactions. Thus, by directly screening for specificity at the interaction network level, our approach is expected to increase the efficacy of action, reduce toxicity and lower the chances for drug resistance. Second, unlike the stable interactions between structured protein domains that have been traditionally difficult to target, the interactions within condensates are weak and highly dynamic making them more readily accessible to small molecules [1]. As a result, condensates are an ideal screening platform for small molecule therapeutics.

In this initial screen, the small molecule hits were tested at 10uM concentration. As we do not yet have dose dependence data, it is possible that the small molecules are effective at lower concentrations. Yet, because these PPIs are highly specific, the small molecule hits are highly selective at affecting chromatin condensates. Given the weak interactions being disrupted within condensates it will be interesting to explore the strength of molecular interactions necessary for drugs to have a noticeable and selective impact on condensates. Our method aims to use core components that drive the biggest differences between chromatin states. However, as in any reconstituted system, we cannot account for all other potential binding factors that may alter chromatin organization. Thus, there is a potential for small molecule hits to interact with off target proteins that are present within the cell but not present within our in vitro condensate. For this reason it is imperative to validate the small molecule hits in human cell lines for selectivity and efficacy, which is outside the scope of the technology brief.

What are the small molecules doing within the chromatin condensate to either disrupt or stabilize a condensate? It is likely that some of the small molecules more generally disrupt or promote weak hydrophobic or electrostatic interactions non-specifically. However, we envision that some of the small molecule hits interact more specifically by altering or stabilizing the conformational states adopted by IDRs. For example, if a given IDR conformational state cooperatively drives condensate formation through multivalent interactions with structured domains, then a small molecule that selectively biases the conformation of the IDR away from this state would drive dissolution of the condensate. Alternatively, a small molecule may selectively bind a transient structured pocket created because of IDR interactions within a given chromatin state. More generally, the small molecule could directly bind to a weak PPI between chromatin and the chromatin protein to either inhibit or enhance the interactions. Because whole chromatin states cannot be structurally resolved with high ehough resolution, traditional structuralbased methods cannot be utilized to identify small molecule binding sites. Therefore, traditional structure activity relationship (SAR) studies using the condensate platform will need to be used to first optimize the potency of the small molecule. Once potency is achieved, binding sites may be identified by fixing its interactions within condensates using chemical crosslinking.

It is known that the networks of interactions that package DNA into chromatin in diseased cells are substantially different than those in healthy cells [5,6]. While there are many chromatin proteins that are promising targets for multiple disease indications, targeting these proteins specifically in diseased and not healthy cells has been challenging. Our platform now enables targeting of these proteins by modeling the unique inter-molecular interactions only found in disease driving chromatin states. The power of this platform lies in leveraging the contextual differences to find chromatin modulators that specifically affect chromatin structure and thus are not limited to traditional inhibitors and agonists. It remains to be seen how well hits from condensate screens translate into therapeutic effects in cell or organismal models of disease. While it is still unclear whether pathological condensate formation is the driver of disease, multiple studies have demonstrated that the IDR interactions in chromatin proteins are critical for maintaining disease phenotypes. In acute myeloid leukemia, the aberrant NUP98-HOXA9 fusion protein contains an IDR that has been shown to promote oncogenesis [24]. This chimera localizes to active enhancer binding sites and active genes to promote long distance looping between enhancers and oncogene promoters, which is associated with condensate formation [24]. Mutations in the IDR disrupts NUP98-HOXA9 condensate formation, reduces NUP98-HOXA9 occupancy on active chromatin and inhibits its long-distance looping [24]. Another example is MeCP2, a key regulator of chromatin compaction. MeCP2 is highly expressed in neurons and contains many IDRs. Mutations within MeCP2 IDR regions lead to RETT syndrome, a neurodevelopment disorder by inhibiting MeCP2-chromatin scaffolding and condensate formation in vitro and in vivo [25]. If the hits from our platform translate into therapeutic effects it would suggest that disease driving protein assemblies can be targeted and that condensates are effective model system for identifying targeting compounds. This would open up a wide new landscape of therapeutic opportunities and ultimately better drugs for patients.

## Figures and Tables

**Table 1:** Results of HP1α chromatin condensate screening. Small molecule hit data classified by condensate imaging phenotype.

Phenotype	Number	Percentage
Mild/no effect	151	47 %
Dissolution of condensates	84	**26 %**
Increased & larger condensates	81	**25 %**
Other interesting phenotypes	4	**.0125 %**
